# Mechanical Stress Induced NOX2 Promotes Endothelial Dysfunction in Ventilator‐Induced Lung Injury: Potential Treatment with Quercetin

**DOI:** 10.1002/advs.202502639

**Published:** 2025-05-20

**Authors:** Tao Jiang, Yabing Zhang, Zhiye Guo, He Ren, Weiyi Hu, Qingping Yao, Yunlong Huo, Yingxin Qi, Kai Huang

**Affiliations:** ^1^ Institute of Mechanobiology & Medical Engineering School of Life Sciences & Biotechnology Shanghai Jiao Tong University 800 Dongchuan Road, Minhang Shanghai 200240 China; ^2^ Key Laboratory for Biomechanics and Mechanobiology of Ministry of Education School of Biological Science and Medical Engineering Beihang University Beijing 100083 China; ^3^ Department of Anesthesiology West China Second University Hospital Sichuan University Chengdu 610041 China; ^4^ Key Laboratory of Birth Defects and Related Diseases of Women and Children Ministry of Education Sichuan University Chengdu 610041 China; ^5^ Key Laboratory for the Genetics of Developmental and Neuropsychiatric Disorders Ministry of Education Shanghai Jiao Tong University 800 Dongchuan Road, Minhang Shanghai 200240 China

**Keywords:** endothelial barrier dysfunction, endothelial cells (ECs), mechanical stress, mechanotransduction, NADPH oxidase (NOX), quercetin, reactive oxygen species (ROS), ventilator‐induced lung injury (VILI)

## Abstract

Mechanical ventilation (MV) is a treatment that helps people who are unable to breathe on their own. However, the use of MV leads to the development of ventilator‐induced lung injury (VILI). Here, it is found that VILI with endothelial barrier disruption is accompanied by elevated reactive oxygen species (ROS) in mice. NADPH oxidase 2 (NOX2, also known as CYBB or gp91phox) is first examined to be the main source of ROS to repress endothelial junction. Besides, 20%‐0.5 Hz high cyclic stretch (high CS) significantly increases NOX2 expression and inhibits endothelial junction protein expression. NOX2 activates the downstream Calcium–calmodulin (CaM)‐dependent protein kinase II (CaMKII)/ Extracellular signal‐regulated protein kinase 1/2 (ERK1/2) signaling pathway through the regulation of ROS. This pathway is confirmed by high MV stimulation in vivo and high CS in vitro. Of note, quercetin, as an antioxidant, is effective in preventing mechanical stretch‐induced endothelial dysfunction by scavenging ROS. Omics data indicated that there is a similar gene expression pattern in cecal ligation and puncture (CLP) and VILI. Pre‐administration of quercetin significantly improves the survival rate of mice via inactivating ROS/CaMKII/ERK1/2 axis. Quercetin pre‐treatment not only preventes MV‐induced lung injury but also attenuates existing inflammation‐induced lung injury.

## Graphical abstract

Abnormal mechanical stretch induced by mechanical ventilation (MV) disrupts endothelial junctions via the NOX2/ROS/CaMKII/ERK1/2 axis both in human aortic endothelial cells (HAECs) in vitro and in ventilator‐induced lung injury (VILI) in mice. Quercetin, an antioxidant predicted to bind to NOX2, can effectively prevent mechanical stretch‐induced endothelial dysfunction by scavenging ROS in vitro and attenuate VILI in vivo.

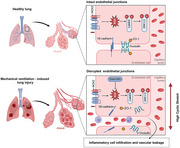

## 1 Introduction

Mechanical ventilation (MV) has become an indispensable therapy in the care of critically ill patients with acute lung injury and acute respiratory distress syndrome (ARDS).^[^ ^1^, ^2^ ^]^ However, MV itself exacerbates lung injury and inflammation which leads to increased mortality in ARDS patients.^[^ ^3^, ^4^ ^]^ Although implementation of a low tidal volume strategy improved outcomes, mortality remains unacceptably high.^[^ ^5^ ^]^ The deleterious effects of MV, known as ventilator‐induced lung injury (VILI), can result from multiple mechanisms, including barotrauma, volutrauma, atelectrauma, and biotrauma.^[^ ^6^ ^]^ Abnormal mechanical stress generated by ventilators is thought to be the predisposing factor to injuries.^[^ ^2^ ^]^ However, the mechanism of mechanical stress generated by MV on cellular mechanotransduction needs to be elucidated. Besides, finding common molecular mechanisms underlying pre‐existing lung injury and the further damage caused by MV will be of great benefit to the prevention and treatment of endothelial dysfunction, vascular remodeling, and lung diseases.

Others and we proved that mechanical stress plays important roles in endothelial dysfunction and vascular remodeling.^[^ ^7^, ^8^, ^9^, ^10^, ^11^, ^12^, ^13^, ^14^ ^]^ Abnormal mechanical stress was associated with vascular remodeling in atherosclerosis,^[^ ^10^, ^13^ ^]^ hypertension,^[^ ^7^, ^11^ ^]^ vein graft failure^[^ ^8^, ^14^ ^]^ and aortic aneurysm formation.^[^ ^15^ ^]^ Mechanical stress‐induced excessive reactive oxygen species (ROS) trigger signaling events that further exacerbate endothelial dysfunction and vascular remodeling.^[^ ^10^, ^16^, ^17^ ^]^ We have shown that ROS produced oxidative stress‐induced endothelial NOS uncoupling and vascular remodeling in the thoracic aortic aneurysm (TAA)^[^ ^18^, ^19^ ^]^ and abdominal aortic aneurysm (AAA).^[^ ^18^, ^19^, ^20^, ^21^ ^]^ We also found that oxidative stress was associated with sepsis‐induced acute lung injury via inducing endothelial cell barrier dysfunction.^[^ ^22^ ^]^ Besides, oxidative stress has also been found to modulate vascular remodeling in hypertension^[^ ^23^ ^]^ and atherosclerosis.^[^ ^24^ ^]^ However, the molecular mechanism by which MV induced high cyclic stretch (high CS) in endothelial dysfunction and lung injury remains unclear, and the role of oxidative stress in this process needs to be elucidated.

Growing evidence indicates that NADPH oxidases (NOXs) are the major source of ROS in vascular diseases.^[^ ^19^, ^20^, ^23^, ^25^, ^26^, ^27^ ^]^ The primary function of NOX is to produce ROS.^[^ ^19^ ^]^ Different NOX isoforms in different cells were found to have specific roles in different vascular diseases.^[^ ^19^ ^]^ Endothelial and fibroblast NADPH oxidase 2 (NOX2, also known as CYBB or gp91^phox^) were involved in the regulation of angiotensin II‐induced hypertension, while NADPH oxidase 1 (NOX1, also known as NOH‐1, MOX1, GP91‐2) in vascular smooth muscle played a more important role than endothelial NOX1 in the development of hypertension.^[^ ^19^, ^23^, ^28^, ^29^, ^30^ ^]^ Notably, NADPH oxidase 4 (NOX4, also known as RENOX, KOX‐1, KOX) appeared to be particularly important in salt‐sensitive hypertension.^[^ ^23^, ^31^, ^32^, ^33^ ^]^ Either NOX1 or NOX2 or both promoted the development of atherosclerosis while NOX4 protected *Apoe* ^−/−^ mice from the formation of atherosclerotic lesions.^[^ ^34^, ^35^, ^36^, ^37^ ^]^ We have proved that NOX1, NOX2 and NOX4 induced ROS promote endothelial dysfunction and aortic aneurysm progress.^[^ ^18^, ^19^, ^20^, ^26^, ^38^ ^]^ However, the role of NOX isoforms in VILI has not explored yet. Therefore, identification of the specific NOX isoforms required for VILI progress will ultimately lead to novel therapies targeting endothelial dysfunction and attenuating VILI.

In the present study, we detected elevated ROS in VILI mice. We identified for the first time that NOX2, but not NOX1 or NOX4, contributes to endothelial dysfunction under abnormal CS in vitro and in the VILI model in vivo. High CS promoted NOX2/Calcium–calmodulin (CaM)‐dependent protein kinase II (CaMKII)/ Extracellular signal‐regulated protein kinase 1/2 (ERK1/2) axis activation induces VILI in vivo and endothelial dysfunction in vitro. Quercetin attenuated high CS‐induced endothelial dysfunction in vitro and lung injury in vivo by scavenging ROS. Besides, quercetin also protects against cecal ligation and puncture (CLP)‐induced acute lung injury. Therefore, ROS activated the CaMKII/ERK1/2 signaling pathway involved both in VILI and CLP‐induced acute lung injury, targeting ROS with quercetin not only prevented high MV‐induced lung injury but also attenuated existing inflammation‐induced lung injury.

## 2 Results

### 2.1 Mechanical Ventilation (MV)‐Induced Lung Injury (VILI) Is Associated with Elevated ROS

**Figure 1:**
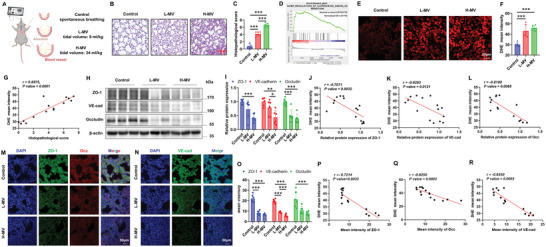
MV‐induced lung injury (VILI) is associated with elevated ROS. A) Schematic representation of the mouse MV model. B,C) Representative H&E images of mice lung tissue sections from the Control, L‐MV, and H‐MV groups (B), and the quantitative analysis of lung injury histological scores (C). Scale bars: 50 µm, *n* = 5–7. D) Enrichment plot of the gene set related to superoxide anion generation in the gene microarray dataset from GSE226807 by Gene set enrichment analysis (GSEA). E,F) Representative DHE fluorescent images (E) and quantitative analysis (F) in mice lung tissue sections from the Control, L‐MV, and H‐MV groups. Scale bars: 20 µm, *n* = 6. G) Correlation analysis of lung injury histological score and DHE mean intensity defined ROS levels indicating that ROS levels accurately reflect the severity of lung injury. R = 0.8975, *p <* 0.0001. *n* = 15. H,I) Representative Western blot (H), and the quantitative analysis (I) of protein expressions of ZO‐1, VE‐cadherin (VE‐cad), and Occludin (Occ) in lung tissue homogenates from the Control, L‐MV, and H‐MV groups. Data are presented as the fold changes relative to the Control group. *n* = 6. J) Correlation analysis of ZO‐1 expression and DHE mean intensity defined ROS levels. R = ‐0.7071, *p* = 0.0032. n = 15. K) Correlation analysis of VE‐cad expression and DHE mean intensity defined ROS levels. R = −0.6283, *p* = 0.0121. *n* = 15. L) Correlation analysis of Occ expression and DHE mean intensity defined ROS levels. R = ‐0.8100, *p* = 0.0085. *n* = 15. M–O) Representative immunofluorescent images of ZO‐1 and Occludin (M) and VE‐cadherin (N), and the quantitative analysis (O) of ZO‐1, Occludin and VE‐cadherin in mice lung tissue sections from the Control, L‐MV and H‐MV groups. Nuclei were counterstained with DAPI. The average fluorescent intensity of ZO‐1, Occludin, and VE‐cadherin was quantified using Image J software. Scale bars: 20 µm, *n* = 6. P) Correlation analysis of ZO‐1 immunofluorescence intensity and DHE mean intensity defined ROS levels. R = −0.7214, *p* = 0.0033. *n* = 15. Q) Correlation analysis of VE‐cad immunofluorescence intensity and DHE mean intensity defined ROS levels. R = −0.8250, *p* = 0.0003. *n* = 15. R) Correlation analysis of Occ immunofluorescence intensity and DHE mean intensity defined ROS levels. R = −0.8250, *p* = 0.0003. *n* = 15. Data are presented as mean ± SD. ^*^ *p* < 0.05, ^**^ *p* < 0.01, ^***^ *p* < 0.001 for the indicated comparisons. L‐MV: low mechanical ventilation; H‐MV: high mechanical ventilation; Occ: Occludin; VE‐cad: VE‐cadherin; DAPI: 4′,6‐diamidino‐2‐phenylindole; GOBP, Gene Ontology Biological Process; ES, enrichment score.

To explore the role of mechanical stress‐induced ROS in VILI, we used a mouse lung injury model caused by MV (**Figure** **1**A). Hematoxylin and eosin (H&E) staining indicated that the lung tissue sections from the L‐MV group and H‐MV group exhibit significant vascular leakage and hemorrhage, inflammatory cell infiltration, and alveolar wall thickening compared to the control group (Figure 1B). The lung histopathological scores of the ventilated lung tissues were gradually increased following the rise of tidal volume (Figure 1C). Based on the gene microarray dataset (GEO accession: GSE226807) from the GEO database, Gene set enrichment analysis (GSEA) revealed a positive correlation between MV and the biological process of superoxide anion (O_2_^−^) generation (http://amigo.geneontology.org/amigo/term/GO:0042554, “GOBP_REGULATION_OF_SUPEROXIDE_ANION_GENERATION”). The enrichment score indicated an upregulated genes set in the MV group which was associated with the regulation of O_2_^−^, the most abundant reactive oxygen species (ROS) within cells (Normalized ES = 1.631782) (Figure 1D). Hence, the roles of oxidative stress in lung injury induced by MV were further investigated. ROS levels in lung tissue sections were assessed using dihydroethidium (DHE) staining. As shown in Figure 1E,F, lung tissues from ventilated mice exhibited a stronger red fluorescent signal from oxidized DHE compared to the control group. Linear correlation between lung histopathological scores and DHE mean intensity was calculated. As shown in Figure 1G, DHE mean intensity defined ROS production accurately predicted lung histopathological scores.

To explore the endothelial barrier function in VILI, we examined the expression of VE‐cadherin, one adhesion junction protein, as well as ZO‐1 and Occludin, two tight junction proteins. Western blot showed a progressive decrease of VE‐cadherin, ZO‐1, and Occludin as tidal volume increased (Figure 1H,I). Of note, reduced ZO‐1, VE‐cadherin, and Occludin levels correlated well with increased ROS production (Figure 1J–L). Immunofluorescence staining of lung tissue sections (Figure 1M–O) also revealed the downregulation of ZO‐1, Occludin, and VE‐cadherin after MV, indicating the disruption of endothelial barrier in VILI. Decreased ZO‐1, VE‐cadherin, and Occludin levels detected by immunofluorescence also correlated well with increased ROS production (Figure 1P–R). These findings suggest that MV elevated ROS levels are associated with endothelial barrier disruption and lung injury in VILI.

### 2.2 NOX2 Elevated ROS Impairs Endothelial Junction under Abnormal Mechanical Stress

**Figure 2:**
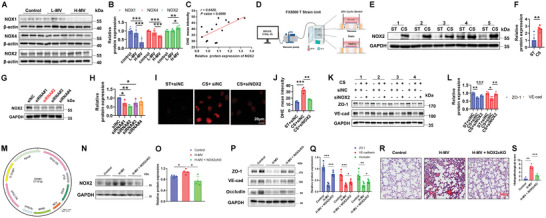
NOX2 elevated ROS impairs endothelial junction under abnormal mechanical stress. A,B) Representative Western blot (A) and the quantitative analysis (B) of protein expressions of NOX1, NOX2, and NOX4 in lung tissue homogenates from the Control, L‐MV, and H‐MV groups. Data are presented as the fold changes relative to the Control group. *n* = 6. C) Correlation analysis of NOX2 expression and DHE mean intensity defined ROS levels indicating that increased NOX2 expression was followed by increased ROS production. R = 0.6420, *p* = 0.0099. *n* = 15. D) Schematic representation of the flexcell FX‐5000 system applied CS to HAECs in vitro. E,F) Representative Western blot (E) and the quantitative analysis (F) of protein expression of NOX2 in HAECs under static and CS conditions. Data are presented as the fold changes relative to the static group. *n* = 5. G,H) Representative Western blot (G) and the quantitative analysis (H) of protein expression of NOX2 in HAECs respectively transfected with five different siRNA fragments (siNC, siRNA#1, siRNA#2, siRNA#3, and siRNA#4). Data are presented as the fold changes relative to the siNC group. *n* = 3. I,J) Representative DHE fluorescent images (I) and the quantitative analysis (J) in HAECs transfected with siNC or siNOX2 under static or CS conditions. Scale bars: 20 µm, *n* = 4. K,L) Representative Western blot (K) and the quantitative analysis (L) of protein expressions of ZO‐1 and VE‐cadherin in HAECs transfected with siNC or siNOX2 under static or CS conditions. Data are presented as the fold changes relative to the siNC group. n = 4. The diagram of RNAi‐custom‐adeno‐associated virus serotype 9 (AAV9‐Cybb‐RNAi) (M). N,O) Representative Western blot (N) and the quantitative analysis (O) of NOX2 expressions. *n* = 4. P,Q) Representative Western blot (P) and the quantitative analysis (Q) of protein expressions of ZO‐1, VE‐cadherin, and Occludin. *n* = 5. R,S) Representative H&E images of mice lung tissue sections from the Control, H‐MV, and H‐MV +NOX2cKO (R), and the quantitative analysis of lung injury histological scores (S). Scale bars: 50 µm, *n* = 5. Data are presented as mean ± SD. ^*^ *p* < 0.05, ^**^ *p* < 0.01, ^***^ *p* < 0.001 for the indicated comparisons. HAEC: human aortic endothelial cells; ST: static; CS: cyclic stretch; NC: negative control; VE‐cad: VE‐cadherin.

In the vascular system, the NOX family is one of the predominant sources of ROS. NOX‐derived ROS also activates other ROS‐generating enzyme systems and further contributes to sustained oxidative stress.^[^ ^19^ ^]^ Since NOX1, NOX2, and NOX4 are expressed in vascular endothelial cells in mice, we first assessed their expressions in VILI and further explored their potential roles in ROS production after MV. Western blot results indicated that NOX2, but not NOX1 and NOX4, was increased following the increase of tidal volume (Figure 2A‐B). Besides, the linear correlation between NOX2 expression and DHE mean intensity was calculated. As shown in **Figure** **2**C, correlation analysis of NOX2 expression and DHE mean intensity defined ROS levels indicating that increased NOX2 expression was followed by increased ROS production. This suggests that NOX2 contributes to elevated ROS levels in ventilated lung tissues (Figure 2A–C).

Excessive local lung dilation is a key factor in lung injury caused by MV.^[^ ^39^ ^]^ To simulate the rhythmic deformation induced by MV with high tidal volume in vivo, 20%‐0.5 Hz CS was applied to HAECs in vitro using the Flexcell‐5000 system (Figure 2D). Compared to the static group, 20% CS significantly increased NOX2 expression in HAECs (Figure 2E,F). NOX2‐targeting siRNA was used to knock down NOX2 expression in HAECs, and the most effective siRNA#2 fragment was selected for the subsequent experiments (Figure 2G,H). We examined whether NOX2 is involved in the regulation of ROS levels and HAEC function in response to CS. As shown in Figure 2I,J, 20% CS remarkably enhanced ROS levels in HAECs, while NOX2 siRNA notably reduced ROS in response to CS. Moreover, western blot analysis showed that 20% CS significantly downregulated the expressions of endothelial junction molecules, i.e., ZO‐1 and VE‐cadherin, which was reversed at least partially by NOX2 siRNA (Figure 2K,L). These results indicated that mechanical stretch increases NOX2 expression which consequently induces ROS production and depresses endothelial junction protein expression.

To consolidate NOX2 as the main factor in VILI, we used RNAi‐custom‐adeno‐associated virus (AAV) to specific knockdown of endothelial NOX2 (NOX2cKO) (Figure 2M) to further verify its role under MV. The data indicated that H‐MV‐induced NOX2 elevation was reversed by NOX2cKO in the lung (Figure 2N,O). Endothelial barrier function was disrupted (downregulation of ZO‐1, VE‐cadherin, and Occludin) by H‐MV, which was however attenuated by NOX2cKO (Figure 2P,Q). Furthermore, histological evaluation by H&E staining indicated significant vascular leakage and hemorrhage, inflammatory cell infiltration, and alveolar wall thickening induced by H‐MV, which was abrogated by NOX2cKO (Figure 2R,S). These data further verified that endothelial NOX2 plays an essential role in promoting acute lung injury induced by MV, and endothelial NOX2 ablation is effective in treating VILI.

### 2.3 Elevated ROS Induce CaMKII/ERK1/2 Signaling Pathway Activation in VILI

**Figure 3:**
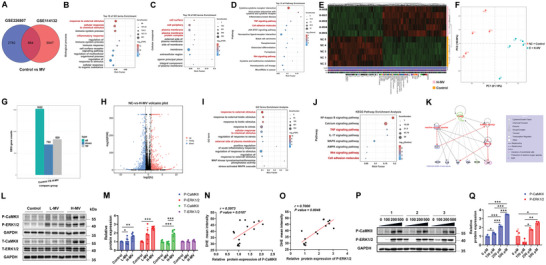
Elevated ROS induce CaMKII/ERK1/2 signaling pathway activation in mechanical ventilation‐induced lung injury (VILI). A) Venn diagram of shared genes in two sets (GSE226807 and GSE114132) of differentially expressed genes in lungs from sham control and MV. B,C) Bubble plot of Gene Ontology (GO) enrichment analysis for 884 genes differentially expressed in both sets. The x‐axis represents the Rich Factor, indicating the degree of enrichment. The color scale indicates the ‐log_2_ (FDR), reflecting the significance of enrichment. Biological process (B) and Cellular component (C). D) Bubble plot of Kyoto Encyclopedia of Genes and Genomes (KEGG) enrichment analysis for 884 genes differentially expressed in both sets. The x‐axis represents the Rich Factor, indicating the degree of enrichment. The color scale indicates the ‐log_2_ (Q‐value), reflecting the significance of enrichment. The differentially expressed genes between H‐MV and control groups E). PCA showed the distribution of the samples in the control group and the H‐MV group F). The downregulated and unregulated genes in H‐MV group compared to control group G). The volcano plot visually displays the distribution of differential genes H). Gene Ontology (GO) analysis between H‐MV group and control group I). KEGG enrichment analysis between H‐MV group and control group J). K) IPA predicts the signaling regulatory network associated with endothelial function, in which the NOX2/ROS/CaMKII/ERK1/2 signaling axis is highlighted in red. L,M) Representative Western blot (L) and the quantitative analysis (M) of phosphorylations and total expressions of CaMKII and ERK1/2 in lung tissue homogenates from the Control, L‐MV, and H‐MV groups respectively. Data are presented as the fold changes relative to the Control group. *n* = 6. N) Correlation analysis of p‐CaMKII expression and DHE mean intensity defined ROS levels indicating that ROS level is positive correlated with p‐CaMKII expression. R = 0.5973, p = 0.0187. *n* = 15. O) Correlation analysis of *p*‐ ERK1/2 expression and DHE mean intensity defined ROS levels indicating that ROS level is positive correlated with ERK1/2 expression. R = 0.7000, p = 0.0048. n = 15. P,Q) Representative Western blot (P) and the quantitative analysis (Q) of phosphorylations of CaMKII and ERK1/2 in HAECs stimulated with H_2_O_2_ at concentrations of 0 µM, 100 µM, 200 µM, and 500 µM respectively. Data are presented as the fold changes relative to the 0 nM group. n = 3. Data are presented as mean ± SD. ^*^ *p* < 0.05, ^**^ *p* < 0.01, ^***^ *p* < 0.001 for the indicated comparisons. L‐MV: low mechanical ventilation; H‐MV: high mechanical ventilation; P‐CaMKII: phosphorylated CaMKII; P‐ERK1/2: phosphorylated ERK1/2; H_2_O_2_: hydrogen peroxide.

The downstream molecular mechanisms by which ROS impaired endothelial integrity were further explored. After analyzing the public transcriptomic data (GSE226807 and GSE114132) of two MV mouse models, 884 genes were screened that differentially expressed (Control versus MV groups) in both models (**Figure** **3**A). Gene Ontology (GO) analysis revealed that these genes significantly enriched cell components related to the cell surface, periphery, and membrane protein complex, and contributed to biological processes including response to external stimulus and inflammatory response (Figure 3B,C). Besides, the Kyoto Encyclopedia of Genes and Genomes (KEGG) enrichment analysis indicated that these differentially expressed genes were significantly enriched in TNF, cell adhesion molecules, and Wnt signaling pathways (Figure 3D). It is worth noting that these signaling pathways are all associated with the multifunctional protein CaMKII,^[^ ^22^, ^40^, ^41^ ^]^ which has been reported to be sensitive to oxidative stress and contribute to endothelial dysfunction in sepsis‐induced acute lung injury.^[^ ^22^ ^]^

Besides, we performed RNA‐seq in control mice and mice subjected to H‐MV (Figure 3E). Principal Component Analysis (PCA showed the distribution of the samples in the control group and the H‐MV group (Figure 3F). There are 702 downregulated and 820 upregulated genes in the H‐MV group compared to the control group (Figure 3G). The volcano plot visually displays the distribution of differential genes (Figure 3H). Gene Ontology (GO) analysis results (Figure 3I) are similar to the public transcriptomic data (GSE226807 and GSE114132). Besides, the differentially expressed genes were also associated with TNF signaling pathways, Wnt signaling pathways, and cell adhesion molecules (Figure 3J). These data indicate that the CaMKII/ERK1/2 axis may play a vital role in lung injury under MV.

Furthermore, network connections between NOX2‐drived ROS and CaMKII/ERK1/2 were predicted using Ingenuity Pathway Analysis (IPA) (Figure 3K). The networks suggested that NOX2 activates the downstream CaMKII/ERK1/2 signaling pathway through the regulation of ROS, ultimately affecting the genes related to endothelial function.

Based on the above bioinformatics analysis, we next investigated the activation of CaMKII and its downstream effectors ERK1/2 in lungs exposed to different levels of MV. As shown in Figure 3L,M, phosphorylation of CaMKII on Thr‐286 residue, which induces its sustained activation,^[^ ^13^ ^]^ was significantly elevated with the increase of tidal volume. The phosphorylation of the downstream target ERK1/2 was also significantly increased, with no change in total ERK1/2 levels. Interestingly, total CaMKII levels also increased significantly in lung tissues exposed to H‐MV compared with the sham control and L‐MV to keep total CaMKII at high level.

To determine whether ROS participates in the activation of the CaMKII/ERK1/2 signaling pathway, a linear correlation between DHE mean intensity and p‐CaMKII or p‐ERK1/2 levels was calculated. As shown in Figure 3N,O, DHE mean intensity defined ROS level was positively correlated with p‐CaMKII level (Figure 3N) and p‐ERK1/2 (3O). Besides, HAECs were treated with a gradient concentration of hydrogen peroxide (H_2_O_2_). The phosphorylations of CaMKII and ERK1/2 were both increased with the rising concentration of H_2_O_2_ (Figure 3P,Q). These results indicate that elevated ROS induced by MV activates the CaMKII/ERK1/2 signaling axis in VILI.

### 2.4 High Cyclic Stretch (High CS) Disrupts Endothelial Junctions via NOX2/ROS/CaMKII/ERK1/2 Axis

**Figure 4:**
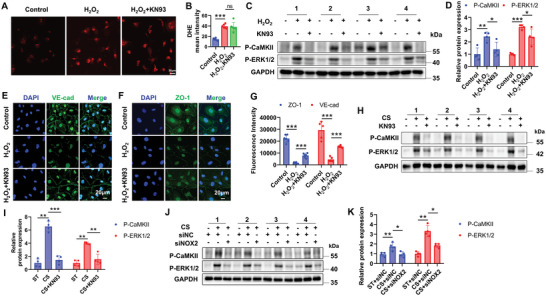
High cyclic stretch disrupts endothelial junctions via NOX2/ROS/CaMKII/ERK1/2 Axis. A,B) Representative DHE fluorescent images (A) and quantitative analysis (B) in control or H_2_O_2_ treated HAECs co‐incubated with or without KN93 (a selective inhibitor for CaMKII). Scale bars: 30 µm. *n* = 6. C,D) Representative Western blot (C) and the quantitative analysis (D) of phosphorylations of CaMKII and ERK1/2 in control or H_2_O_2_ treated HAECs co‐incubated with or without KN93. Data are presented as the fold changes relative to the Control group. *n* = 4. E–G) Representative immunofluorescent images of VE‐cadherin (E) and ZO‐1 (F) and the quantitative analysis (G) of ZO‐1 and VE‐cadherin in control or H_2_O_2_ treated HAECs co‐incubated with or without KN93. Nuclei are counterstained with DAPI. Scale bars: 20 µm. The fluorescence intensity of ZO‐1 and VE‐cadherin is quantified using Image J software. *n* = 5–6. H,I) Representative Western blot (H) and the quantitative analysis (I) of phosphorylations of CaMKII and ERK1/2 in HAECs pre‐treated with or without KN93 under static or CS conditions. Data are presented as the fold changes relative to the static group. *n* = 4. J,K) Representative Western blot (J) and the quantitative analysis (K) of phosphorylations of CaMKII and ERK1/2 in HAECs transfected with siNC or siNOX2 under static and CS conditions. Data are presented as the fold changes relative to the ST + siNC group. *n* = 4. Data are presented as mean ± SD. ^*^ *p* < 0.05, ^**^ *p* < 0.01, ^***^ *p* < 0.001 for the indicated comparisons. H_2_O_2_: hydrogen peroxide; P‐CaMKII: phosphorylated CaMKII; P‐ERK1/2: phosphorylated ERK1/2; VE‐cad: VE‐cadherin; DAPI: 4′,6‐diamidino‐2‐phenylindole; ST: static; CS: cyclic stretch; NC: negative control.

To verify the regulatory role of the NOX2/ROS/CaMKII/ERK1/2 axis in endothelial function under CS, CaMKII inhibitor KN93 and NOX2 siRNA were used. We found that KN93 had no effect on DHE intensity after H_2_O_2_ treatment (**Figure** **4**A,B), which suggests that CaMKII was not upstream of ROS production. Western blot results showed that H_2_O_2_ treatment significantly increased the phosphorylation of CaMKII and ERK1/2 in HAECs compared to the control group, while KN93 effectively inactivated CaMKII/ERK1/2 pathway (Figure 4C,D). Furthermore, KN93 effectively alleviated H_2_O_2_‐induced suppression of endothelial junction molecules, i.e., ZO‐1 and VE‐cadherin (Figure 4E,F,G).

To confirm the role of mechanical stretch in modulating the CaMKII/ERK1/2 pathway, we pre‐incubated HAECs with KN93 before being subject to CS. Western blot results showed that CS significantly increased the phosphorylation of CaMKII and ERK1/2, while KN93 effectively blocked this effect (Figure 4H,I). Additionally, NOX2 knockdown significantly inhibited the activation of the ROS/CaMKII/ERK1/2 signaling pathway induced by CS (Figure 4J,K). These findings indicate that mechanical stretch activates the NOX2/ROS/CaMKII/ERK1/2 signaling axis, leading to the disruption of endothelial junctions.

### 2.5 Quercetin Effectively Prevents Mechanical Stretch‐Induced Endothelial Dysfunction by Scavenging ROS In Vitro

**Figure 5:**
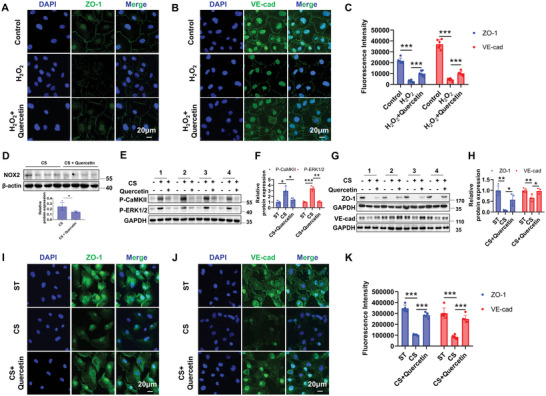
Quercetin effectively prevents mechanical stretch‐induced endothelial dysfunction by scavenging ROS in vitro. A–C) Representative immunofluorescent images of ZO‐1 (A) and VE‐cadherin (B) and the quantitative analysis (C) in control or H_2_O_2_ treated HAECs co‐incubated with or without quercetin. Nuclei are counterstained with DAPI. Scale bars: 20 µm. The fluorescence intensity of ZO‐1 and VE‐cadherin is quantified using Image J software. *n* = 6. Representative Western blot and the quantitative analysis of NOX2 (D). *n* = 4. E,F) Representative Western blot (E) and the quantitative analysis (F) of phosphorylations of CaMKII and ERK1/2 in HAECs pre‐treated with or without quercetin under static or CS. Data are presented as the fold changes relative to the static group. *n* = 4. G,H) Representative Western blot (G) and the quantitative analysis (H) of protein expressions of ZO‐1 and VE‐cadherin in HAECs pre‐treated with or without quercetin under static or CS. Data are presented as the fold changes relative to the static group. *n* = 4. I–K) Representative immunofluorescent images of ZO‐1 (I) and VE‐cadherin (J) and the quantitative analysis (K) in HAECs pre‐incubated with or without quercetin under static or CS. Nuclei are counterstained with DAPI. Scale bars: 20 µm. The fluorescence intensity of ZO‐1 and VE‐cadherin is quantified using Image J software. *n* = 5‐6. Data are presented as mean ± SD. ^*^ *p* < 0.05, ^**^ *p* < 0.01, ^***^ *p* < 0.001 for the indicated comparisons. L‐MV: low mechanical ventilation; H‐MV: high mechanical ventilation; P‐CaMKII: phosphorylated CaMKII; P‐ERK1/2: phosphorylated ERK1/2; VE‐cad: VE‐cadherin; H_2_O_2_: hydrogen peroxide; DAPI: 4′,6‐diamidino‐2‐phenylindole.

The potential role of antioxidants in attenuating endothelial dysfunction in vitro under CS was further explored. Immunofluorescence results showed that quercetin effectively alleviates H_2_O_2_‐induced disruption of endothelial junctions (**Figure** **5**A–C). HAECs subjected to CS were pre‐incubated with quercetin (1 µM) to explore the role of quercetin during mechanical stretch application. NOX2 was decreased by quercetin under CS (Figure 5D). As shown in Figure 5E,F, quercetin significantly inhibited CS‐induced activation of CaMKII and ERK1/2. In addition, quercetin restored CS‐induced suppression of ZO‐1 and VE‐cadherin levels (Figure 5G,H). Immunofluorescence data further confirmed that CS‐induced ZO‐1 and VE‐cadherin expression reduction was reversed by quercetin (Figure 5I‐K). These findings indicate that quercetin significantly alleviates endothelial dysfunction induced by mechanical stretch, which provides a promising preventive effect in vivo.

### 2.6 Antioxidant Drug Quercetin Effectively Attenuates VILI In Vivo

**Figure 6:**
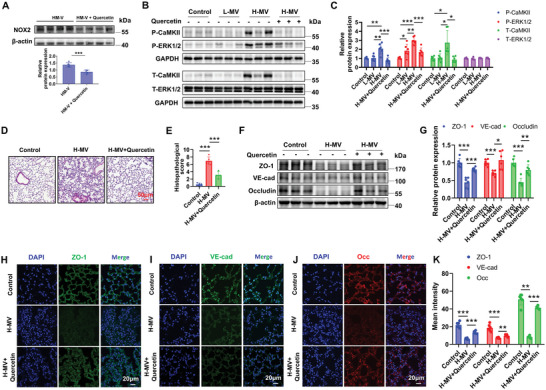
Antioxidant drug quercetin effectively attenuates ventilation‐induced lung injury (VILI). A. Representative Western blot, and the quantitative analysis of NOX2 in lung tissue homogenates from the H‐MV groups pre‐treated with or without quercetin respectively. *n* = 5. B,C) Representative Western blot (B), and the quantitative analysis (C) of phosphorylations and total expressions of CaMKII and ERK1/2 in lung tissue homogenates from the Control, L‐MV, and H‐MV groups pre‐treated with or without quercetin respectively. Data are presented as the fold changes relative to the Control group. *n* = 6. D,E) Representative H&E images of mice lung tissue sections from the Control, L‐MV, and H‐MV groups pre‐treated with or without quercetin (D), and the quantitative analysis of lung injury histological scores (E). Scale bars: 50 µm, *n* = 4–5. F,G) Representative Western blot (F), and the quantitative analysis (G) of protein expressions of ZO‐1, VE‐cadherin and Occludin in lung tissue homogenates from the Control, L‐MV, and H‐MV groups pre‐treated with or without quercetin. Data are presented as the fold changes relative to the Control group. *n* = 6. H–K) Representative immunofluorescent images of ZO‐1 (H), VE‐cadherin (I), and Occludin (J) and the quantitative analysis (K) of ZO‐1, VE‐cadherin, and Occludin in mice lung tissue sections from the Control, L‐MV and H‐MV groups pretreated with or without quercetin. Nuclei are counterstained with DAPI. The average fluorescent intensity of ZO‐1, Occludin and VE‐adherin is quantified using Image J software. Scale bars: 20 µm, *n* = 6. Data are presented as mean ± SD. ^*^ *p* < 0.05, ^**^ *p* < 0.01, ^***^ *p* < 0.001 for the indicated comparisons. L‐MV: low mechanical ventilation; H‐MV: high mechanical ventilation; P‐CaMKII: phosphorylated CaMKII; P‐ERK1/2: phosphorylated ERK1/2; VE‐cad: VE‐cadherin; DAPI: 4′,6‐diamidino‐2‐phenylindole.

To evaluate the therapeutic effect of quercetin at the animal level, mice in the H‐MV group were pre‐treatment with quercetin (15 mg kg^−1^ day^−1^) three days prior to MV. NOX2 was significantly reduced by quercetin under H‐MV (**Figure** **6**A). Besides, Quercetin effectively decreased the elevation of p‐CaMKII and p‐ERK1/2 induced by H‐MV, accompanied by significant repression of total CaMKII (Figure 6B,C). Pathohistological analysis showed that quercetin significantly reduced vascular leakage and hemorrhage, inflammatory cell infiltration, and alveolar wall thickening, with a significantly decreased pathohistological score of lung injury (Figure 6D,E). Consistent with the cellular experiments in vitro, quercetin robustly elevated the protein expressions of ZO‐1, VE‐cadherin, and Occludin in mice treated with H‐MV (Figure 6F,G). Furthermore, immunofluorescence staining of lung tissue sections revealed the restoration of these three endothelial junction molecules in drug‐treated mice compared to the H‐MV alone group (Figure 6H–K). These findings indicate that quercetin significantly represses the activation of the CaMKII/ERK1/2 signaling pathway by scavenging ROS, which effectively attenuates VILI.

### 2.7 Quercetin Markedly Improves Survival and Alleviates Acute Lung Injury in Cecal Ligation Puncture (CLP)‐Induced Septic Mice

**Figure 7:**
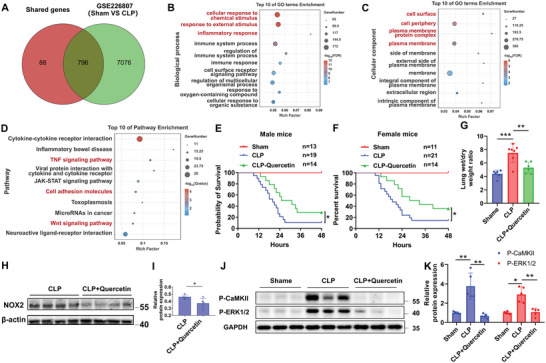
Quercetin markedly improves survival and alleviates acute lung injury in cecal ligation puncture (CLP)‐induced septic mice. A) Venn diagram of shared genes between 884 genes in Figure 3. A and the differentially expressed genes in lungs between normal control and CLP (GSE226807). B,C) Bubble plot of Gene Ontology (GO) enrichment analysis for 796 genes differentially expressed in both sets. The x‐axis represents the Rich Factor, indicating the degree of enrichment. The color scale indicates the ‐log2 (FDR), reflecting the significance of enrichment. D) Bubble plot of Kyoto Encyclopedia of Genes and Genomes (KEGG) enrichment analysis for 796 genes differentially expressed in both sets. The x‐axis represents the Rich Factor, indicating the degree of enrichment. The color scale indicates the ‐log_2_ (Q‐value), reflecting the significance of enrichment. E,F) Survival curves of male (E) and female (F) mice pre‐treated with or without quercetin in the sham and CLP groups. G) Quantitative analysis of lung wet‐to‐dry weight ratio in the Control and CLP groups. *n* = 6. Representative Western blot (H) and quantitative analysis of NOX2 (I) from CLP model pretreated with or without quercetin. n = 5. J,K) Representative Western blot images (J) and quantitative analysis (K) of phosphorylations of CaMKII and ERK1/2 in lung tissue homogenates from the Control and CLP groups pretreated with or without quercetin. Data are presented as the fold changes relative to the shame group. *n* = 5. Data are presented as mean ± SD. ^*^ *p* < 0.05, ^**^ *p* < 0.01, ^***^ *p* < 0.001 for the indicated comparisons. P‐CaMKII: phosphorylated CaMKII; P‐ERK1/2: phosphorylated ERK1/2; CLP: cecal ligation puncture.

In addition to exploring the role of quercetin in VILI, we also studied the effect of quercetin in treating pre‐existing lung damage caused by inflammation. Omics data revealed that 90.05% (796 out of 884) of the 884 genes identified in Figure 3A were also differentially expressed in the CLP model compared to the sham group (**Figure** **7**A) indicating that there may be a common pathological molecular mechanism in VILI and CLP‐induced lung injury. GO and KEGG analyses showed the cellular components, biological processes, and pathways enriched of the 796 genes that differentially expressed both in VILI and CLP (Figure 7B‐D). These results suggest a similar gene expression pattern activated during VILI and CLP. Therefore, we also evaluated the therapeutic effect of quercetin in the CLP mouse model. Interestingly, pre‐treatment of quercetin significantly improved the survival rate both in male (Figure 7E) and female (Figure 7F) mice. Compared to the sham group, the CLP group showed a significant increase in lung wet‐to‐dry weight ratio, which was effectively alleviated by quercetin (Figure 7G). The expression of NOX2 was decreased by quercetin in the CLP model (Figure 7H‐I). The phosphorylation levels of CaMKII and ERK1/2 were increased in the CLP group and were remarkably reversed by quercetin (Figure 7J‐K), this is consistent with the results in VILI (Figure 6B‐C). Therefore, quercetin not only prevented VILI but also attenuated inflammation‐induced lung injury.

### 2.8 Molecular Docking Reveals the Potential Interaction between Quercetin and NOX2

**Figure 8:**
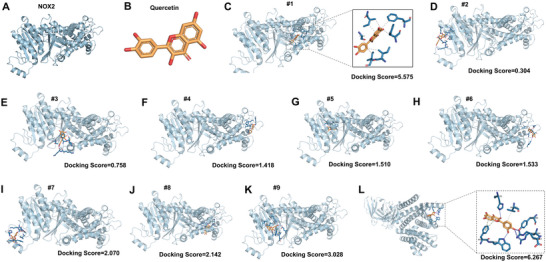
Molecular docking reveals potential interaction between quercetin and NOX2. A) The 3D structure of NOX2 protein retrieved from the AlphaFold Protein Structure Database. **B**. The 3D structure of quercetin obtained from the PubChem database. C–K) Molecular docking simulations were performed using the DeepMice platform, with quercetin docked into 9 predicted active centers of NOX2. Docking scores ranged from 0.304 to 5.575, with the highest docking score observed at pocket #1 (Docking Score = 5.575). The binding interactions and poses were visualized using PyMOL (Version 3.0.4), and the results from the highest docking score were analyzed. L) Molecular docking simulations were performed using the DeepMice platform, with quercetin docked into human NOX2 core complex structure resolved by cryogenic electron microscopy (cryo‐EM).

NOX2 conformation and ligand‐induced structural rearrangements affect its activity and ROS production.^[^ ^42^, ^43^ ^]^ In addition to acting as an antioxidant, we also explored whether quercetin could interact directly with NOX2. The 3D structure of the NOX2 protein (**Figure** **8**A) was retrieved from the AlphaFold Protein Structure Database while the 3D structure of quercetin was obtained from the PubChem database (Figure 8B). Pocket region detection identified 9 potential ligand‐binding regions of NOX2 with the DeepMice platform (http://www.deepmice.com/). Quercetin was docked at each of these 9 regions, yielding 9 protein‐ligand complex models (Figure 8C–K). The highest docking score was observed at region 1 (Docking Score = 5.575), indicating that quercetin exhibited the strongest binding affinity at this pocket on NOX2 (Figure 8C). Of note, we further employed cryo‐EM to resolve the human NOX2 core complex structure reported by Noreng et al.^[^ ^43^ ^]^ for additional molecular docking simulations. The docking score of 6.627 confirmed a potential interaction between quercetin and NOX2 (Figure 8L), which is consistent with the findings from the above docking analysis. These findings suggest that quercetin may directly interact with NOX2, thereby modulating its ROS‐generating function.

## 3 Discussion

This study is the first to elucidate the molecular mechanism by which endothelial NOX2 also known as cytochrome b(558) subunit beta, cytochrome b‐245 heavy chain or gp91phox)‐dependent ROS production promotes VILI (Graphic abstract). Endothelial dysfunction was induced by NOX2/ROS/CaMKII/ERK1/2 signaling pathway both in the VILI model in vivo and in cultured cells under high CS in vitro. Intriguingly, oral administration of quercetin attenuates both H‐MV‐induced lung injury and CLP‐induced lung injury via inactivating ROS/CaMKII/ERK1/2 axis. Therefore, targeting NOX2 could be a novel therapeutic strategy for the treatment of VILI, and scavenging ROS may not only prevent high MV‐induced lung injury but also alleviate existing inflammation‐induced lung injury.

In the mammalian lung, alveoli and capillaries form a continuous tight barrier that facilitates gas exchange, while endothelial cells (ECs) form the sides of the capillaries.^[^ ^44^ ^]^ ECs in pulmonary capillaries sense dynamic mechanical forces generated by the physiological respiratory cycle, and the MV with inappropriate parameters can directly cause mechanical damage to ECs, thereby causing lung injury.^[^ ^45^ ^]^ Therefore, targeting endothelial dysfunction could be an effective way of treating VILI. Current research on endothelial mechanotransduction in VILI has mainly focused on mechanosensitive receptors such as ion channels and integrins.^[^ ^46^, ^47^, ^48^ ^]^ Transient receptor potential vanilloid 4 (TRPV4) promotes acute calcium‐dependent permeability during VILI in isolated mouse lungs.^[^ ^46^ ^]^ Endothelium‐specific knockout of piezo‐type mechanosensitive ion channel component 1 (Piezo1) induces the pathogenesis of VILI.^[^ ^47^ ^]^ Integrin alphavbeta5 modulates pulmonary endothelial barrier function and lung vascular permeability by interacting with the actin cytoskeleton in VILI.^[^ ^48^ ^]^ However, the roles of NOX isoforms in VILI have not investigated yet. Here, we found for the first time that NOX2 was activated by high CS in ECs to produce ROS that led to endothelial dysfunction and VILI. Importantly, inhibition of NOX2 was an effective approach to alleviate endothelial dysfunction under high CS.

NOX2 is the first member of the NOX family of enzymes to be discovered.^[^ ^19^ ^]^ In the cardiovascular system, NOX2 is expressed in ECs, adventitial fibroblasts, and cardiomyocytes to produce superoxide directly.^[^ ^19^ ^]^ The role of NOX2 has been explored in many cardiovascular diseases including hypertension,^[^ ^29^, ^49^ ^]^ aortic aneurysms,^[^ ^50^, ^51^ ^]^ atherosclerosis.^[^ ^52^, ^53^ ^]^ Angiotensin II (ANG II)‐induced aortic superoxide production and blood‐pressure elevation was alleviated by Nox2 knockout in mice.^[^ ^49^ ^]^ Endothelial NOX2 overexpression exacerbated blood pressure in the ANG II infusion model.^[^ ^29^ ^]^ Besides, NOX2 is upregulated in abdominal aortic aneurysms (AAA) in patients.^[^ ^50^ ^]^ Nox2 deletion prevents AAA formation in ANG II‐infused hph‐1 mice.^[^ ^20^, ^51^ ^]^ In atherosclerosis, NOX2 ablation reduced aortic superoxide production, induced NO bioavailability, and attenuated lesion formation^[^ ^52^ ^]^ while endothelial NOX2 overexpression did not further accelerate atherosclerosis progress even though the superoxide production and macrophage recruitment were increased in Apoe^−/−^ mice.^[^ ^53^ ^]^ Here, we found that NOX2 is activated after H‐MV, inducing excessive ROS and disrupting endothelial junctions. However, NOX1 and NOX4 were all decreased under this condition. This may be a compensatory response of the body, but this compensatory effect cannot offset the damage caused by NOX2 activation. The compensation among NOX families has also been found in another study. In the cataract study, Das et al. found that Nox2 upregulation compensates for a loss of Nox4 expression in vivo, and loss of Nox4 also led to the compensatory upregulation of the essential Nox2 subunit p47phox.^[^ ^54^ ^]^

Neuromuscular blockers, neuromuscular‐blocking drugs, or neuromuscular blocking agents (NMBAs) are used to block the transmission at the neuromuscular junction to achieve skeletal muscle relaxation.^[^ ^55^ ^]^ The mechanism and clinical application of quercetin are different from NMBAs. Quercetin, as a bioactive flavonoid compound, has clinical significance reflected in its multiple pharmacological effects, such as anti‐inflammatory, antioxidant, antitumor, and cardiovascular protection.^[^ ^56^ ^]^ Quercetin decreases macrophage pyroptosis by activating NRF2 through competitively binding to the Arg483 site of Kelch‐like ECH‐associated protein 1 to alleviate atherosclerosis.^[^ ^57^ ^]^ Quercetin and its derivatives exert their antioxidation and anti‐inflammation effects via PI3K/AKT/NF‐κB, Nrf2/ARE, and MAPK pathways to attenuate sepsis‐associated diseases.^[^ ^58^ ^]^ Quercetin could also act as a ferroptosis inhibitor to provide a therapeutic strategy for treating diseases related to ferroptosis including acute kidney injury.^[^ ^59^ ^]^ Besides, quercetin also prevents neuronal damage by promoting mitophagy and inhibiting mtROS‐mediated NLRP3 inflammasome activation in microglia, which provides a potential way for treating neuroinflammation‐related diseases. Of note, the role of quercetin in VILI and related molecular mechanisms has not been studied yet. In this study, we found that high CS‐induced ROS play a vital role in endothelial dysfunction in VILI. Quercetin was found to have antioxidant and anti‐inflammatory effects in several pathological conditions, so we explored its role in VILI. Our results indicated that the high CS‐activated NOX2/ROS/CaMKII/ERK1/2 pathway is inhibited by quercetin both in vitro and in vivo. Besides, our data verified that quercetin also attenuates sepsis‐induced lung injury both in males and females. Therefore, quercetin pre‐treatment not only prevented high MV‐induced lung injury but also attenuated existing inflammation‐induced lung injury. Notably, the AlphaFold Protein Structure Database predicted the 3D structures of NOX2 (AlphaFold NOX2) and cryo‐EM resolved the human NOX2 core complex structure (cryo‐EM NOX2).^[^ ^60^ ^]^ Molecular docking simulation results indicated that both AlphaFold NOX2 and cryo‐EM NOX2 retain the binding sites with quercetin. These predictions indicate that quercetin may directly interact with NOX2, potentially altering its spatial conformation and thereby modulating its ROS‐generating function. Since quercetin is a health supplement, it could be rapidly translated into clinical practice to revolutionize the management of the MV.

## 4 Experimental Section

### Animal Studies

Male C57BL/6J mice, aged 8–12 weeks, were purchased from Beijing Vital River Laboratory Animal Technology Co., Ltd (Beijing, China). The mice were maintained under specific pathogen‐free conditions. All mice were randomly assigned to control and experimental groups. All animal experiments were approved by the Animal Ethics Committee of Shanghai Jiao Tong University and the Experimental Animal Management and Ethics Committee of West China Second University Hospital, Sichuan University and in accordance with the Animal Management Rules of China (55, 2001, Ministry of Health, China).

### Animal Model Construction and Tissue Collection

The mechanical ventilation (MV) model was performed as described,^[^ ^61^, ^62^ ^]^ mice were anesthetized via intraperitoneal injection of ketamine. The animals were positioned supine on a heating pad to maintain body temperature. For the ventilation procedure, a 20G intravenous catheter was used for oral tracheal intubation and subsequently connected to a small animal ventilator (Model’683’, Harvard Apparatus, MA, USA). The experimental groups and ventilator parameters were as follows: low MV (L‐MV) with tidal volume 9 mL kg^−1^, respiratory rate 160 breaths min^−1^, and I:E ratio of 1:1; high MV (H‐MV) with tidal volume 34 mL kg^−1^, respiratory rate 70 breaths min^−1^, and I:E ratio of 1:1. For the H‐MV + drug treatment group, mice were pretreated with the antioxidant drug quercetin (15 mg kg^−1^ day^−1^) for three days prior to being subjected to MV using the same ventilator parameters as in the H‐MV group. Mice in the non‐ventilated control group were allowed to breathe spontaneously. The duration of ventilation was set to 4 h for all groups.

The cecal ligation puncture (CLP) model was constructed as previously described.^[^ ^22^ ^]^ Mice were fasted for 12 h prior to surgery with free access to water. Anesthesia was maintained using a gas anesthesia system (Matrx VIP 3000) with 5% isoflurane at a flow rate of 1.5 L min^−1^. After skin disinfection, a 1 cm midline abdominal incision was made to expose the cecum. The cecum was ligated at the middle position between the colon base and the cecal end using a 4‐0 braided suture, followed by a puncture with a 21‐gauge needle to induce infection. Finally, the cecum was returned to the abdomen and the incision was closed. For the sham operation group, mice underwent the same skin incision and abdominal exposure without ligation and puncture, followed by the closure of the incision. Mice were euthanized 16 h post‐operation for tissue collection unless specified otherwise for survival curve analysis.

For tissue collection, 50 µL of heparin was injected into the right ventricle, followed by perfusion with 3 mL PBS to flush the pulmonary vasculature. Lungs were quickly excised, and washed in cold PBS, and connective tissues were cleaned on ice. The left lung was fixed in 4% paraformaldehyde and then embedded in paraffin for histological analysis, while the right upper lung lobe was embedded in optimal cutting temperature (OCT) compound, rapidly frozen in liquid nitrogen, and sectioned into 5 µm slices for dihydroethidium (DHE) staining or immunofluorescence staining. The remaining lung lobes were frozen at −80 °C for Western blot analysis.

### H&E Staining and Histological Analysis

The left lung lobe was fixed in 4% paraformaldehyde for 24 h, then washed with PBS, dehydrated, and embedded in paraffin. The tissue was sectioned into 5 µm slices, deparaffinized, rehydrated, and stained with hematoxylin and eosin (H&E). Slides were examined under a microscope for imaging and histological analysis. Lung injury pathology scores were assessed as previously described.^[^ ^22^ ^]^ Three random fields (200x magnification) per slide were observed and analyzed for each sample. Each sample was scored for vascular leakage and hemorrhage, inflammatory cell infiltration, and alveolar wall thickening. Each feature was scored on a scale from 0 to 4 points, where 0 indicates no injury within the field of view, 1 indicates injury affecting less than 25% of the area, 2 indicates 25–50% injury, 3 indicates 50–75% injury, and 4 indicates 75–100% injury. The sum of these three scores was taken as the comprehensive lung injury score for each mouse, with 0 indicating no lung injury and 12 indicating maximum lung injury. The scoring was conducted in a blinded manner.

### DHE Staining

Frozen lung tissue sections embedded in OCT were incubated with freshly prepared DHE (Cat#: D7008, Sigma Aldrich) in the dark at room temperature for 30 min. Sections were washed three times with PBS, mounted with coverslips, and imaged using a fluorescence microscope (IX71, Olympus). Fluorescence intensity was quantified using Image J software. Additionally, Confocal laser scanning microscopy (Fluoview 1000, Olympus) was used for Z‐stack imaging and maximum intensity projections were created using Image J.

### Western Blot

For lung tissue samples, 20–30 mg of lung tissue was homogenized in 300–450 µL RIPA lysis buffer with protease inhibitors using a pre‐cooled tissue grinder (75 Hz, 90 s). The homogenate was centrifuged at 12,000 g for 10 min at 4 °C, and the supernatant was collected as total protein. For cell samples, cells were lysed on ice with RIPA lysis buffer with protease inhibitors for 10 min, followed by centrifugation at 12 000 g for 10 min at 4 °C to remove cell debris, and the supernatant was collected as total protein. Proteins (20–40 µg) were separated by SDS‐PAGE and transferred to PVDF membranes, blocked with 5% non‐fat milk for 1 h at room temperature, and incubated with primary antibodies overnight at 4 °C. The following primary antibodies were used: NOX1 (1:1000, Cat#: NBP1‐31546, Novus), NOX2 (1:1000, Cat#: NBP2‐41291, Novus), NOX4 (1:1000, Cat#: NB110‐58849, Novus), Zonula occludin 1 (ZO‐1) (1:1000, Cat#: 61–7300, Invitrogen), Occludin (1:1000, Cat#: 33–1500, Invitrogen), VE‐cadherin (1:1000, Cat#: V1514‐200UL, Sigma‐Aldrich); P‐CaMKII (1:1000, Cat#: SC‐32289, Santa Cruz Biotechnology Inc), CaMKII (1:1000, Cat#: SC‐5306, Santa Cruz Biotechnology Inc), P‐ERK1/2 (1:1000, Cat#: 4370, Cell Signaling Technology, Danvers, MA, USA), ERK1/2 (1:1000, Cat#: 4695, Cell Signaling Technology), GAPDH(1:2000, Cat#: 60004‐1‐Ig, Proteintech), β‐actin(1;2000, Cat#: 2876, Proteintech). The next day, membranes were then incubated with HRP‐conjugated secondary antibodies (anti‐rabbit IgG (1:3000, Cat#: L3012, Signalway Antibody), anti‐mouse IgG (1:3000, Cat#: L3032, Signalway Antibody) for 1 h at room temperature, and signals were detected using the Quantity One chemiluminescence imaging system (BioRad). Image J software was used for quantitative analysis.

### Immunofluorescence Imaging

Frozen lung tissue sections were fixed in ice‐cold acetone for 15 min and permeabilized with 0.25% Triton X‐100 for 20 min. Cell samples were fixed with 4% paraformaldehyde for 10 min and permeabilized with 0.25% Triton X‐100 for 10 min. Both tissue and cell samples were blocked with 10% goat serum for 1 h at room temperature and incubated with primary antibodies overnight at 4 °C. The following primary antibodies were used: ZO‐1(1: 200, Cat#: 61–7300, Invitrogen), Occludin (1: 200, Cat#: 33–1500, Invitrogen), VE‐cadherin(1:200, Cat#: V1514‐200UL, Sigma‐Aldrich). The next day, sections were incubated with fluorescent secondary antibodies and DAPI for 1 h at room temperature. After mounting with coverlips, the samples were imaged using a confocal laser scanning microscope (Fluoview 1000, Olympus). The fluorescence signals stemming from the nucleus were eliminated while performing fluorescence intensity analysis with ImageJ software to ensure that the fluorescence intensity values obtained accurately depict the integrity of the endothelial junctions being studied.

### Cyclic Stretch (CS) Loading

Human aortic endothelial cells (HAECs) were seeded on gelatin‐coated flexible silicone membranes (FlexCell International, USA) for 24 h. CS loading was performed using the FX5000 T Strain Unit (FlexCell International, USA) to simulate cell deformation during H‐MV in vivo, with 20% stretch amplitude and 0.5 Hz frequency for 4 h.^[^ ^63^, ^64^ ^]^ Control cells were cultured under the same conditions without CS. Cells were collected immediately after CS for protein extraction.

### Cell Culture and NOX2 siRNA Transfection

HAECs were cultured in complete endothelial cell medium with supplements, fetal bovine serum, and penicillin‐streptomycin at 37 °C in a humidified atmosphere with 5% CO2. For siRNA transfection, cells were passaged into six‐well plates when they reached 80% confluence. Once the cells reached 50% confluence in six‐well plates, they were transfected with human NOX2 siRNA (GenePharma) or negative control siRNA (GenePharma) using the siRNA‐Mate transfection reagent (Cat#: G04002, GenePharma) according to the manufacturer's instructions. Briefly, 3.75 µL of 20 µM siRNA stock solution was mixed with 42.5 µL of Buffer, and gently pipetted to mix, forming the siRNA pre‐mix. Fifteen µL of Plus transfection reagent was added to the above solution, and the mixture was immediately pipetted to obtain the siRNA/Plus complexes. This siRNA transfection complex was then added to the cell culture medium to achieve a final siRNA concentration of 37.5 nM, and the mixture was gently swirled to ensure even distribution of the siRNA. Transfection efficiency was assessed by Western blot 60 h post‐transfection. The siRNA sequences are listed in Table S1 (Supporting Information).

### RNAi‐Custom‐Adeno‐Associated Virus to Specific Knockdown of Endothelial NOX2 (NOX2cKO)

RNAi‐custom‐adeno‐associated virus called AAV9‐Cybb‐RNAi(P24M0087) was constructed by Shanghai Genechem Co., Ltd for specific knockdown of endothelial NOX2 (NOX2cKO). Following the package, AAV‐9 was administrated via intratracheal injection on day 1 (30 µL, 1.0 × 7.04E + 12 v.g. mL^−1^). Four weeks later, the mice were sacrificed and the lung tissues were harvested for further detection.

### Transcriptome Analysis

Transcriptome data were downloaded from the GEO database for reanalysis. Microarray gene expression profile data were obtained from GSE226807, selecting the sequencing data from the Control group, MV group, and CLP group. Differential gene expression was analyzed between pairs of groups: Sham versus CLP and Control versus MV, using the online tool GEO2R (https://www.ncbi.nlm.nih.gov/geo/info/geo2r.html#references). High‐throughput RNA‐seq data were obtained from GSE114132, and differential expression analysis was conducted using the edgeR^[^ ^65^ ^]^ tool in the online data analysis platform OmicShare (www.OmicShare.com/tools). The screening criteria for differentially expressed genes (DEGs) among the three groups were as follows: │log2FC│>1, adjusted p<0.05. Subsequently, common differentially expressed genes among the three datasets were identified and Venn diagrams were drawn using OmicShare tools (www.OmicShare.com/tools). Differentially expressed genes were then subjected to Kyoto Encyclopedia of Genes and Genomes (KEGG) enrichment analysis and Gene Ontology (GO) enrichment analysis, and the results were visualized using Enrichment analysis bubble charts.

### RNA Sequencing

The lung tissues from the control and H‐MV groups were harvested and sent to Shanghai Genechem Co., Ltd. for RNA sequencing (*n* = 5 for each group). The clustering of the index‐coded samples was performed on a cBot Cluster Generation System using TruSeq PE Cluster Kit v3‐cBot‐HS (Illumia) according to the manufacturer's instructions. After cluster generation, the library preparations were sequenced on an Illumina Novaseq platform and 150 bp paired‐end reads were generated. After quality control, reads mapping to the reference genome, and quantification of gene expression level, differential expression analysis was performed using the DESeq2 R package (1.16.1). Principal Component Analysis (PCA) was used to assess differences between groups and duplication of samples within groups. GO enrichment analysis and KEGG enrichment analysis were performed as shown in *Transcriptome analysis*.

### Ingenuity Pathway Analysis (IPA)

IPA software (Version 111 725 566, Qiagen, Germany) integrates the available knowledge on genes, drugs, chemicals, protein families, processes, and pathways based on the interactions and functions derived from the Ingenuity Pathways Knowledge Database Literature, and understands the complex biological and chemical systems at the core of life science research‐based on lectures or predicated analysis.^[^ ^66^ ^]^ In the IPA (Qiagen, Germany) system, vascular endothelial function‐related gene sets were identified, including Permeability of microvascular endothelial cells, Cell adhesion of microvascular endothelial cells, and Endothelial barrier function of vascular endothelial cells. These three gene sets, comprising a total of 59 genes, were added to a New My Pathway along with NOX2 (CYBB). NOX2 was categorized into Set A, while the functional gene sets were categorized into Set B. Using the Build/Path Explorer in IPA, eight potential molecules involved in the regulation of microvascular endothelial cell barrier function and potentially regulated by NOX2 were identified. These eight molecules were further validated and screened using Overlay/Diseases and Functions/Interaction of endothelial cells and Overlay/Diseases and Functions/Production of ROS. Ultimately, six molecules highly associated with the regulation of endothelial barrier function by NOX2 were identified. The key regulatory nodes (ROS, ERK1/2) were displayed in the regulatory network. Finally, CAMKII (CAMK2) was added to the pathway, and the Build/Connect tool in IPA was used to elucidate the potential regulatory relationships among NOX2, CaMKII, ROS, and ERK1/2.

### Cell Treatment

Immortalized HAECs were ordered from QuiCell (Shanghai, China) and cultured in an endothelial cell culture medium (QuiCell‐Pri‐8003). HAECs were treated with various reagents post‐seeding in six‐well plates or cell culture slides. To explore the effects of ROS on cell function, HAECs were treated with different concentrations of hydrogen peroxide (H_2_O_2_) (0, 100 µM, 200 µM, 500 µM)^[^ ^67^, ^68^, ^69^ ^]^ for 24 h before protein collection; For CaMKII inhibition, HAECs were co‐incubated with H_2_O_2_ (500 µM) and the CaMKII inhibitor KN93^[^ ^70^, ^71^ ^]^ (1 µM, Cat#: 139298‐40‐1, MCE) for 24 h; For ROS scavenging, the antioxidant drug quercetin (1 µM) was co‐incubated with H_2_O_2_hydrogen peroxide (500 µM) for 24 h. For experiments related to CS loading, HAECs were pre‐treated with KN93 (1 µM) or quercetin (1 µM) and then subjected to mechanical loading. Subsequently, the cells were collected for protein detection or fluorescence staining.

### Survival Curve

For survival curve analysis, the survival status of mice was observed every 30 min starting 12 h post‐CLP surgery to record the time of natural death after CLP initiation. Survival curves were analyzed using Prism software and presented as Kaplan‐Meier plots.

### Lung Wet/Dry Weight Ratio

The lung wet/dry weight ratio was used as an indicator of lung edema, reflecting increased endothelial permeability and the severity of lung injury.^[^ ^22^, ^63^ ^]^ Lungs were weighed immediately after excision (wet weight), then dried in an oven at 60 °C for 48 h and re‐weighed (dry weight). The ratio of wet to dry weight was calculated.

### Potential Interaction between Quercetin and NOX2

Protein and ligand preparation: The 3D structure of NOX2 protein was obtained from the AlphaFold Protein Structure Database(https://alphafold.ebi.ac.uk/) or PDB (PDB Entry – 7U8G) in PDB format. The 3D structure of the ligand, quercetin, was retrieved from the PubChem database (https://pubchem.ncbi.nlm.nih.gov/) in SDF format. The quercetin SDF file was then converted to MOL2 format using Open Babel (version 3.1.1), a chemical toolbox designed to interconvert chemical file formats and prepare molecules for molecular docking. Besides, the cryogenic electron microscopy (cryo‐EM) human NOX2 core complex structure resolved by Noreng et al^[^ ^60^ ^]^ was used for additional molecular docking simulations. Molecular docking simulation: Molecular docking of NOX2 and quercetin was performed using the DeepMice platform (http://www.deepmice.com/), an advanced tool for molecular interaction and binding affinity prediction. Potential binding sites were identified through pocket detection, then molecular docking simulations were conducted at the predicted active centers to evaluate the binding mode of quercetin. Visualization of docking results: The docking results were visualized using PyMOL (Version 3.0.4), a molecular visualization system. By visualizing the complex structure obtained from docking, the spatial conformation and key sites of the interaction between quercetin and NOX2 proteins were predicted and analyzed.

### Data Analysis

All statistical analyses were performed using GraphPad Prism (Version 9.5.0, GraphPad Software, San Diego, CA, USA). Data are presented as mean ± standard deviation (mean ± SD), with all experiments conducted at least three times. All data were first assessed for normality using the Shapiro‐Wilk test. When *P* > 0.05, data were considered normally distributed, and parametric tests were employed. For comparisons between two groups, a paired t‐test was used for paired experimental groups. For unpaired experimental groups, an F‐test was conducted to assess the equality of variances. An unpaired t‐test was applied to data with equal variances, while an unpaired t‐test with Welch's correction was used for data with unequal variances. For comparisons among three or more groups, repeated measures one‐way ANOVA (RM one‐way ANOVA) was used to analyze data differences for paired experimental groups, followed by Tukey's post‐hoc test for specific group comparisons. For unpaired experimental groups, the Brown‐Forsythe test was used to assess variance equality. Ordinary one‐way ANOVA was applied to data with equal variances, followed by Tukey's multiple comparisons test. For data with unequal variances, Brown‐Forsythe and Welch ANOVA tests were performed, followed by Dunnett's T3 multiple comparisons test. In correlation analysis, the normality of the data was first assessed using the Shapiro‐Wilk test. For normally distributed data, Pearson's correlation coefficient (r) was applied to evaluate the strength of associations. For non‐normally distributed data, Spearman's rank correlation coefficient (ρ) was used. For comparisons between two groups, the Mann‐Whitney test was employed, and for comparisons among three or more groups, the Kruskal‐Wallis test was used, followed by Dunn's post‐hoc test to correct for multiple comparisons. *P* < 0.05 was regarded as statistically significant. Throughout the figures, ^*^, ^**^, and ^***^ indicated *p* values < 0.05, 0.01, and 0.001, respectively.

## Conflict of Interest

The authors declare no conflict of interest.

## Supporting information



Supporting Information

## Data Availability

The data that support the findings of this study are available from the corresponding author upon reasonable request.
