# Supplementary material for: Mechanical Stress Induced NOX2 Promotes Endothelial Dysfunction in Ventilator‐Induced Lung Injury: Potential Treatment with Quercetin
Source: Adv Sci (Weinh). 2025 May 20;12(28):2502639. doi: 10.1002/advs.202502639 (PMC12302586; doi:10.1002/advs.202502639)
Supplement: Supplementary file 1 — Supporting Information [file ADVS-12-2502639-s001.docx]

**Table S1 Sequences used in gene-specific siRNA sense strands.**

| siRNA Name | Antisense Sequence (5′-3′) |
| --- | --- |
| siNC | ACGUGACACGUUCGGAGAATT |
| siNox2_1 | UUUCUCCUCAUCAUGGUGCTT |
| siNox2_2 | UUCAAAGUAAGACCUCCGGTT |
| siNox2_3 | UCUUUAUUCUCUUUCGAGCTT |
| siNox2_4 | UAUAUAGCUACUCCUUGGUTT |
